# Proteomic prediction of aortic disease: from statistical association to biological insight

**DOI:** 10.1097/JS9.0000000000003175

**Published:** 2025-08-06

**Authors:** Han Yan, Jiayi Chen, Linghua Pei

**Affiliations:** a The Second Clinical Medical College, Zhejiang Chinese Medical University, Hangzhou, China; b The Second Affiliated Hospital of Zhejiang Chinese Medicine University, Hangzhou, China

*Dear Editor,*

We have read with great interest the article by Li *et al* on the proteomic prediction of aortic aneurysm and dissection (AA/AD)^[1]^. Their work represents a commendable advance, identifying a four-protein signature (CST3, MMP12, MEGF9, and CXCL17) that predicts future events up to a decade before clinical diagnosis. While these findings are promising, we wish to offer four perspectives that may enrich their interpretation and guide future research in this area.

Firstly, the decision to use a composite endpoint, while a pragmatic choice to increase statistical power, warrants careful consideration. This approach risks masking crucial biological heterogeneity between aortic aneurysms and dissections. The identified signature—particularly the strong signal from MMP12, a key enzyme in atherosclerotic matrix degradation—is likely biased towards the more prevalent abdominal aortic aneurysm (AAA) subtype^[2]^. To advance precision medicine, future analyses that disaggregate these endpoints to uncover pathology-specific biomarkers would be invaluable.

Secondly, while the discovery of early protein elevation based on a single baseline measurement is a significant finding, the predictive power of a single time-point assessment may be surpassed by the protein’s dynamic trajectory. Future longitudinal studies incorporating serial proteomic measurements are essential to determine if the rate of change, rather than the absolute level, is a more potent predictor. This would enable a critical shift from static risk assessment to dynamic patient monitoring^[3]^.

Thirdly, the study’s findings prompt an important question regarding the tissue origin of this circulating proteomic signature. Plasma proteomics cannot distinguish whether these biomarkers originate from the diseased aortic wall itself or from systemic processes. This distinction is critical, as a locally-derived marker would suggest a direct therapeutic target, whereas a systemic one might be a non-causal indicator or a confounder. Integrating tissue-level ‘omics with plasma data represents a crucial next step to validate the local relevance of these markers.

Finally, the biological heterogeneity within the final protein signature offers an important, yet unexplored, insight. The panel’s predictive power appears to derive from its synergy between CST3, an indicator of systemic inflammation and renal stress^[4]^, and MMP12, a key mediator of local aortic pathology^[2]^. This synergy suggests a potential “two-hit” disease model, where systemic susceptibility and local vulnerability converge to maximize risk. Future research focused on this interplay could provide a more nuanced understanding of AA/AD pathogenesis.

In conclusion, while the study by Li *et al* provides a valuable exploratory foundation, we believe that addressing these perspectives on endpoint specificity, temporal dynamics, tissue origin, and biological heterogeneity is crucial. Doing so will be essential for translating this promising statistical association into a robust, biologically-informed, and clinically actionable tool for patient risk stratification. (We ensure this article is compliant with the TITAN Guidelines^[5]^.)

## Data Availability

Not applicable.
